# Quality Evaluation of *Juniperus rigida* Sieb. et Zucc. Based on Phenolic Profiles, Bioactivity, and HPLC Fingerprint Combined with Chemometrics

**DOI:** 10.3389/fphar.2017.00198

**Published:** 2017-04-19

**Authors:** Zehua Liu, Dongmei Wang, Dengwu Li, Shuai Zhang

**Affiliations:** Department of Forestry Engineering, College of Forestry, Northwest A&F UniversityYangling, China

**Keywords:** *Juniperus rigida*, phenolic profiles, bioactivities, HPLC fingerprint, chemometrics

## Abstract

*Juniperus rigida* (*J. rigida*) which is endemic to East Asia, has traditionally been used as an ethnomedicinal plant in China. This study was undertaken to evaluate the quality of *J. rigida* samples derived from 11 primary regions in China. Ten phenolic compounds were simultaneously quantified using reversed-phase high-performance liquid chromatography (RP-HPLC), and chlorogenic acid, catechin, podophyllotoxin, and amentoflavone were found to be the main compounds in *J. rigida* needles, with the highest contents detected for catechin and podophyllotoxin. *J. rigida* from Jilin (S9, S10) and Liaoning (S11) exhibited the highest contents of phenolic profiles (total phenolics, total flavonoids and 10 phenolic compounds) and the strongest antioxidant and antibacterial activities, followed by Shaanxi (S2, S3). A similarity analysis (SA) demonstrated substantial similarities in fingerprint chromatograms, from which 14 common peaks were selected. The similarity values varied from 0.85 to 0.98. Chemometrics techniques, including hierarchical cluster analysis (HCA), principal component analysis (PCA), and discriminant analysis (DA), were further applied to facilitate accurate classification and quantification of the *J. rigida* samples derived from the 11 regions. The results supported HPLC data showing that all *J. rigida* samples exhibit considerable variations in phenolic profiles, and the samples were further clustered into three major groups coincident with their geographical regions of origin. In addition, two discriminant functions with a 100% discrimination ratio were constructed to further distinguish and classify samples with unknown membership on the basis of eigenvalues to allow optimal discrimination among the groups. Our comprehensive findings on matching phenolic profiles and bioactivities along with data from fingerprint chromatograms with chemometrics provide an effective tool for screening and quality evaluation of *J. rigida* and related medicinal preparations.

## Introduction

The evergreen coniferous shrub, *Juniperus rigida*, is endemic to East Asia, and mainly distributed in the northern hemisphere in cold temperate regions of North and Northeastern China, Korea, and Japan. The berries of *J. rigida* have been widely used in Korean traditional medicine to treat rheumatoid arthritis and dropsy (Lee et al., 2010) and its branches and leaves utilized in traditional Mongolian medicine in China and as an important raw material to provide essential oil for the chemical industry (Wei and Shibamoto, 2007; Wu and Lin, 2014; Abdel et al., 2015). Chemical and pharmacological research on *J. rigida* has led to the discovery of bioactive components, such as flavonoids and phenolic compounds that potentially contribute to its antioxidant activity (Robards et al., 1999; Woo et al., 2011; Taviano et al., 2013). Moreover, lignans and flavonoids from *J. rigida*, such as catechin, podophyllotoxin, and amentoflavone, exert strong biological effects, including anti-inflammatory, anticancer, and antiviral activities (Lee et al., 2008; Gordien et al., 2009; Ryu et al., 2010; Lesjak et al., 2011, 2013, 2014; Jeong et al., 2012).

Despite the widespread application and commercial importance of *Juniperus*-derived products, evaluation of *J. rigida* has traditionally been based on morphological characteristics resulting from natural genetic development (e.g., mutation, recombination) of individual species with little consideration of phytochemical components and bioactivities (Li et al., 2014; Liu Q. X. et al., 2016; Sobierajska et al., 2016). These shortcomings have limited the utilization of *J. rigida*, highlighting the urgent necessity for a simple and flexible approach for quality evaluation.

Chromatographic fingerprint analysis is a widely accepted technique due to its high confidence in quality assessment of agricultural products (EMEA London, 1999; SFDA Beijing, 2000; US Food Drug Administration Rockville, 2000; WHO Geneva, 2000). Chemometric methods, in particular, hierarchical cluster analysis (HCA) and principle component analysis (PCA) that involve the objective collection of chemical profiles, are widely applied in analyzing information from chemical analyses of herbal medicines (Yan et al., 2001; Zhang et al., 2005; Wan et al., 2006; Shi et al., 2008; Wang et al., 2014; Liu W. et al., 2016). However, to our knowledge, fingerprint analysis has not been applied to evaluate the quality of *J. rigida* to date. In the current study, high-performance liquid chromatography (HPLC) fingerprint associated with chemometrics methods was employed to assess the quality of *J. rigida*, with a view to optimizing its utility in healthcare and food industries.

The main objectives of this study were to: (1) analyze the variations in phenolic profiles and bioactivities of *J. rigida* derived from 11 regions in China, (2) establish an effective method to evaluate the quality of *J. rigida* based on HPLC fingerprint analysis with subsequent HCA, PCA, and discrimination analysis (DA), and (3) provide a theoretical basis for quality evaluation of *J. rigida* and related medicinal preparations.

## Materials and methods

### Plant materials

**Figure 1:**
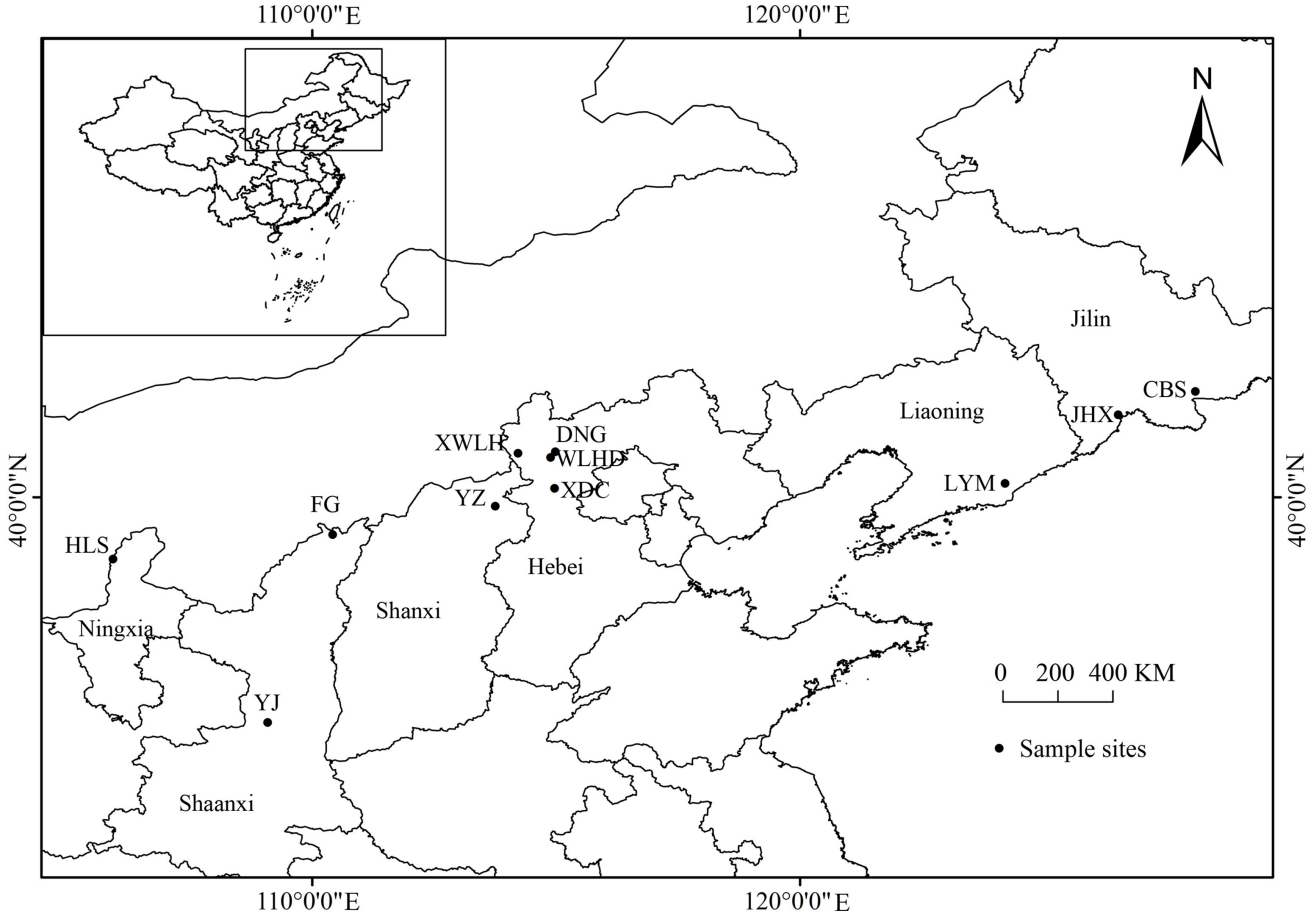
**Location of ***J. rigida*** samples collected from different regions**. Maps were generated using ArcGIS 10.0 (ESRI Inc., 2014).

Needles of *J. rigida* were collected from 11 regions covering all primary origins in China (Table 1, Figure 1). The voucher specimen was identified by the professor Dengwu Li and was deposited at Herbarium of the Northwest A&F University, Yangling, China. All materials were air-dried, powdered, and stored in the dark at −20°C until further analysis.

**Table 1:** *****J. rigida*** samples collected from different regions of China**.

**No**.	**Origin**	**Code**	**Coordinates**	**Altitude (m)**	**Number of samples**	**Group**
S1	Helan Mountain, National nature reserve, NX	HLS	E105°55′N38°44′	2090	5	3
S2	Yulin, Fugu, Dachanghan, SHX	FG	E110°25′N39°14′	1200	5	1
S3	Tongchuan, Yaozhou, Yijun, SHX	YJ	E109°05′N35°23′	1190	5	1
S4	Datong, Hunyuan, SX	YZ	E113°45′N39°49′	1750	5	3
S5	Zhangjiakou, Chongli, HB	DNG	E114°59′N40°56′	1100	5	3
S6	Zhangjiakou, Dabaozhen, HB	XDC	E114°58′N40°11′	1400	5	3
S7	Zhangjiakou, Xiaosuangou, HB	XWLH	E114°13′N40°54′	1050	5	3
S8	Zhangjiakou, Wulahada, HB	WLHD	E114°53′N40°49′	1050	5	3
S9	Baishan, Jinhua, JL	JHX	E126°31′N41°41′	660	5	1
S10	Changbai Mountain, National nature reserve, JL	CBS	E128°06′N42°10′	1146	5	2
S11	Dandong, Fengcheng, LN	LYM	E124°12′N40°17′	228	5	2

### Instrumentation and reagents

A HPLC analysis was conducted with an Agilent Series 1260 liquid chromatograph equipped with a quaternary gradient pump system and variable-wavelength detector system connected to a reversed-phase (RP) SB-C 18 column (5 μm, 4.6 × 250 mm, Agilent, USA). Data collection was performed using ChemStation software (Agilent, USA). Chromatograph-grade methanol and acetonitrile were purchased from Sigma-Aldrich Co. Ltd (St. Louis, Missouri, USA).

Folin-Ciocalteus's phenol reagent (Beijing Solarbio Co. Ltd, PR China), 1,1-diphenyl-2-picrylhydrazyl (DPPH), 2,2-azino-bis (3-ethyl-benzothiazoline-6-sulphonic acid) diammonium salt (ABTS), 2,4,6-Tripyridyl-s-triazine (TPTZ), and 6-Hydroxy-2,5,7,8-tetramethylchroman-2-carboxylic acid (Trolox, Sigma-Aldrich Co., St. Louis, Missouri, USA), gallic acid, quercetin, and acetic acid (Tianjin Bodi Chemical Holding Co. Ltd, PR China) were used. All solutions were filtered through 0.22 mm nylon filters before use. Reagents were of analytical grade and dissolved in deionized water (18 MΩ cm).

### Preparation of crude extracts

The extraction process was optimized using a response surface method considering the impact of various factors. Each powdered sample was treated as described in the optimized extraction process: extraction time (1 h), aqueous ethanol concentration (60%), extraction temperature (70°C), number of extractions (2), liquid-solid ratio (20:1). Filtrates were concentrated via rotary evaporation under vacuum to obtain the crude extracts and stored at −20°C in the dark until further use. All extractions were conducted in triplicate.

### Microbial strains and culture

Antimicrobial activity was tested against nine bacterial strains provided by the Microbial Culture Collection Center of Guangdong Institute of Microbiology, China. Tests against four gram-positive bacteria (*Staphylococcus aurous Rosen Bach* ATCC6538, *Pseudomonas aeruginosa* ATCC29212, *Bacillus subtilis* ATCC6633, and *Listeria monocytogenes* ATCC19115) and five gram-negative bacteria (*Klebsiella pneumoniae* ATCC46117, *Salmonella enteritidis* ATCC14028, *Salmonella typhimurium* CMCC50115, *Salmonella paratyphi* CMCC50093, and *Escherichia coli* ATCC25922) were conducted. All strains were cultured at 37°C on Mueller-Hinton medium.

### Determination of total phenolic content (TPC) and flavonoid content (TFC)

The Folin-Ciocalteau colorimetric method was adopted to determine the TPC, with minor modifications (Burcu et al., 2014; Mocan et al., 2014). The phenolic content of all samples was calculated from the gallic acid equivalent based on a calibration curve of gallic acid standard solutions (10, 20, 40, 60, 80, 100, 200, 300, and 400 μg/mL). All values were expressed as millimole gallic acid equivalent per 100 g of dry weight (mmol equiv. GAE/100 g). Data were expressed as means ± *SD* for three replicates.

The TFC was determined using the sodium borohydride/chloranil-based (SBC) assay developed by He et al. (2008). Sample solutions were diluted to a concentration of 20 mg/mL. A calibration curve was generated using different concentrations of quercetin (0.1–10.0 mM). The total flavonoid content of ethanol extracts of needles of *J. rigida* was expressed as millimoles of quercetin equivalents per 100 g dry weight (mmol equiv. QUE/100 g). Data were expressed as means± *SD* for three replicates.

### Determination of antioxidant activity of *J. rigida*

The DPPH radical scavenging capacity was measured using the method described by Yen and Chen (1995) with some modifications. Trolox was used as the positive control, and all samples were tested in triplicate. A lower IC_50_ value indicates higher DPPH radical scavenging activity.

The scavenging effects for the ABTS^•+^ radical cation were monitored as described in the published literature, with some modifications (Yang et al., 2013; Mocan et al., 2015). Trolox and phosphate-buffered saline (PBS) solutions were used as the standard antioxidant and blank samples, respectively. Data were expressed in terms of micromoles of trolox equivalents per g dry extract weight (μmol eq. trolox/g). All determinations were performed in triplicate.

FRAP activity was determined using the protocol of Benzie and Strain (2008) with some modifications. Trolox was employed as the standard solution and FRAP results were expressed in terms of micromoles Trolox equivalent per gram dry extract weight (mmol equiv. Trolox/ g). All experiments were performed in triplicate.

### Determination of antibacterial activity of *J. rigida*

#### Disc-diffusion method

The antibacterial activity of phenolic extracts was evaluated via the paper disc agar diffusion method reported by Özer et al. (2007), with some modifications. The test solution was prepared by dissolving 5 mg/mL crude extracts in dimethyl sulfoxide (DMSO). Penicillin, tetracycline, and chloramphenicol (10 μg/mL) were used as positive controls and DMSO solution as the negative control. The zone of inhibition was measured in millimeters. The diameters of the inhibition zone (DIZ) were ranked as follows: not sensitive (−) for zone diameters equal to 8 mm or below, sensitive (+) for zone diameters between 8 and 14 mm, very sensitive (++) for zone diameters between 14 and 20 mm (Schroeder and Messing, 1949; Ponce et al., 2003). Inhibition zones were calculated as the average values from triplicate experiments.

#### Minimum inhibitory concentration (MIC) and minimum bactericidal concentration (MBC)

MIC and MBC were determined according to the agar dilution method, with minor modifications (Silva et al., 2011; Burcu et al., 2014; Mocan et al., 2014). Briefly, the extract was dissolved in DMSO, and 1.5 mL stock solution incorporated into 13.5 mL Mueller Hinton broth to obtain concentrations of 5, 2.5, 1.25, 0.625, 0.3125, and 0.1563 mg/mL. MIC was taken from the lowest concentration well that visually showed no growth after 24 h while MBC was the lowest concentration showing no visible growth after 48 h. Tests were performed in triplicate to confirm the reproducibility of the results.

### Determination of individual compounds of *J. rigida* needles via reversed-phase HPLC analysis

#### RP-HPLC analysis

All sample solutions were filtered through 0.22-mm nylon filters before use and separated via RP-HPLC to obtain chromatograms. The mobile phase consisted of water with 0.5% acetic acid (solvent A) and acetonitrile (solvent B). Flow rate was maintained at 0.8 mL/min and the gradient elution program set as follows: 5% B (0 min), 20% B (0–20 min), 25% B (20–35 min), 40% B (35–40 min), 65% B (40–55 min), 80% B (55–60 min), 100% B (60–70 min). The injection volume was 20 μL and the detection wavelength was 254 nm. Experiments were carried out in triplicate.

#### Validation of the HPLC procedure

The HPLC fingerprint assay was adopted to establish a standard for quality evaluation of *J. rigida* (Wan et al., 2006; Wang et al., 2007; Shi et al., 2008). In a previous study, our group validated the HPLC procedure via analysis of precision, reproducibility and repeatability Liu W. et al. (2016). All relative standard deviation (RSD) of the relative peak areas were <3%, suggesting that the conditions for fingerprint analysis were optimal.

### Statistical analysis

As recommended by the Chinese pharmacopeia Committee, data analysis was performed using Computer Aided Similarity Evaluation System (CASES) software. The correlation coefficients of entire chromatographic patterns among samples were calculated and simulated mean chromatograms as well as characteristic peaks generated using CASES. Based on the correlation coefficient (median), the software was also used to conduct similarity analysis (SA) of different chromatograms. HCA, PCA, and DA were performed using SPSS software (SPSS for Windows 19.0, SPSS Inc., USA) (Duan et al., 2012). HCA was conducted to classify samples with regard to similarities of chemical properties, and the “average linkage between groups” method and cosine applied in the measurements. Three principal components obtained using PCA were applied to assess similarities and differences among the samples (Nyambaka and Ryley, 2004; Pierce et al., 2006). DA analysis facilitated the identification and assortment among the groups and confirmed the results of SA, HCA, and PCA, improving quality evaluation of *J. rigida* more acceptable.

All measurements were carried out in triplicate, and the results presented as mean values ± *SD* (standard deviation). Statistical analysis was performed via one-way analysis of variance (ANOVA) followed by Duncan's test. *P* < 0.05 were considered significant. Data were analyzed using SPSS 19.0 (SPSS Inc., Chicago, IL, USA) for Windows and figures generated using SigmaPlot 12.0 and Photoshop 8.0 for Windows.

## Results and discussion

### Total phenolic and flavonoid contents of *J. rigida* needles from different regions

Among *J. rigida* derived from the 11 districts, the total phenolic and total flavonoid contents varied significantly (Table 2). Total phenolic compounds were the most abundant in Fengcheng (S11) with contents of 31.64 mmol equiv. GAE/100 g, and lowest in Dabaozhen (S6) with contents of 13.34 mmol equiv. GAE/100 g. Consistent with total phenolic content data, the highest and lowest total flavonoid contents were detected in S11 and S6 (84.53 mmol equiv. QUE/100 g, 39.05 mmol equiv. QUE/100 g, respectively). Overall, abundance of total phenolic and flavonoid contents of *J. rigida* was in the order: Fengcheng (S11), Changbai Mountain (S10), Yijun (S3), Fugu (S2), Chongli (S5), Xiaosuangou (S7), Jinhua (S9), Datong (S4), Wulahada (S8), Helan Mountain (S1), Dabaozhen (S6). Therefore, among the diverse *J. rigida* samples, those from Fengcheng and Changbai Mountain were of the highest quality due to their rich phenolic contents. Variations in the total phenolic and flavonoid contents in *J. rigida* needles were potentially due to differences in the levels, properties, and proportions of their active ingredients, which, in turn, may be attributed to distribution in diverse geological zones, leading to different therapeutic properties of the same species from various growth regions.

**Table 2:** **Total phenolic and flavonoid contents in phenolic extracts of ***J. rigida*** needles from different origins**.

**Origin**	**Total phenolic content (mmol equiv. GAE/100 g)**	**Total flavonoid content (mmol equiv. QUE/100 g)**
S1	17.06 ± 0.89^d^	47.54 ± 3.95^ef^
S2	25.07 ± 1.83^b^	66.97 ± 9.16^c^
S3	25.81 ± 1.91^b^	64.39 ± 6.31^cd^
S4	18.63 ± 1.62^d^	60.56 ± 6.24^d^
S5	24.70 ± 2.03^b^	47.64 ± 2.31^ef^
S6	13.34 ± 1.09^e^	39.05 ± 3.23^f^
S7	21.68 ± 2.01^c^	70.82 ± 3.21^bc^
S8	17.26 ± 0.96^d^	48.51 ± 4.03^e^
S9	19.62 ± 0.98^cd^	46.43 ± 2.12^ef^
S10	26.53 ± 2.76^b^	76.22 ± 4.23^ab^
S11	31.64 ± 1.26^a^	84.53 ± 5.03^a^

*Data (means ± SD, n = 3) within a row with different superscripts are significantly different (p < 0.05)*.

### Phenolic compounds of *J. rigida* needles

**Figure 2:**
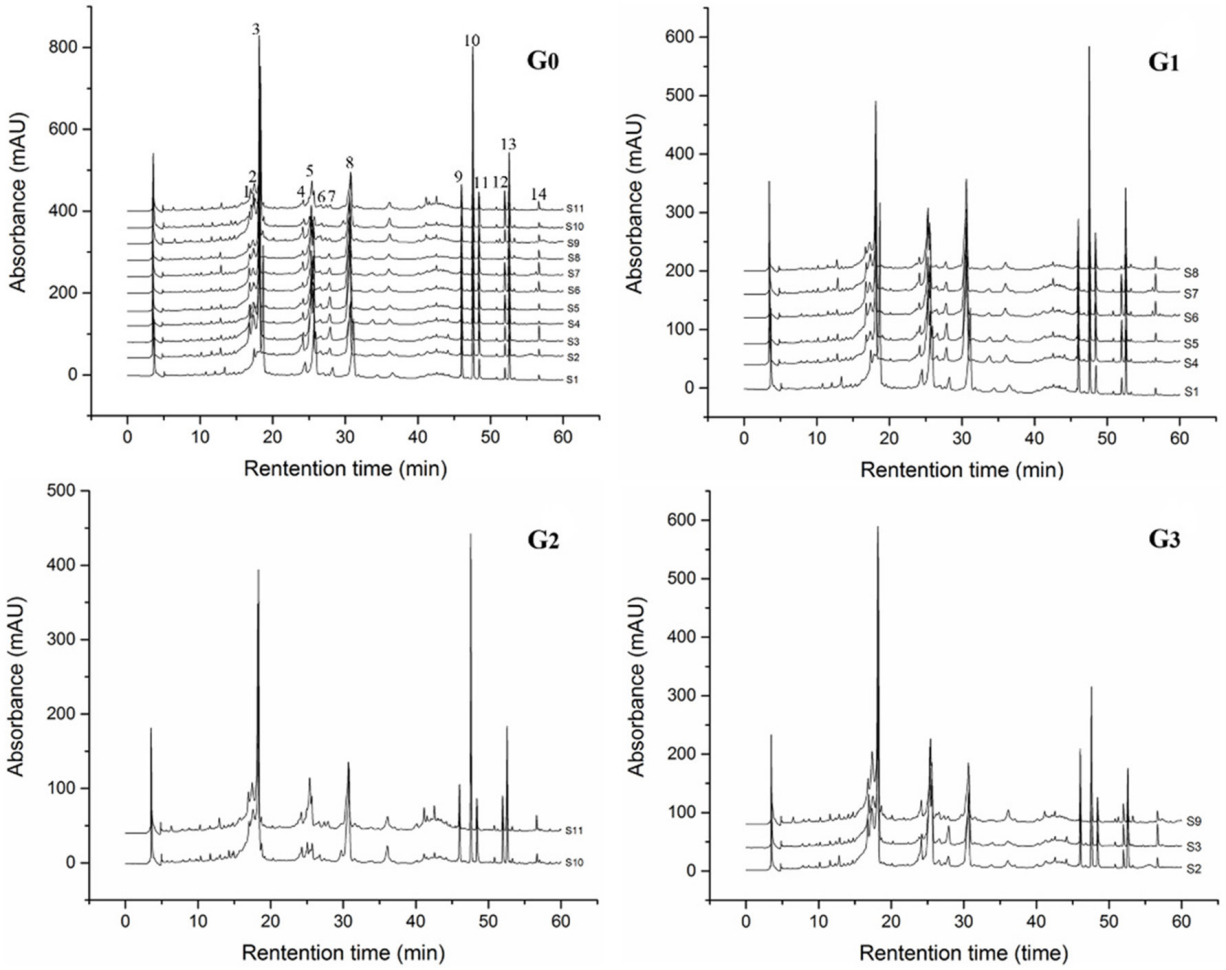
**HPLC fingerprinting profiles (G0) and visual assortment of ***J. rigida*** from different regions (G1, G2, G3)**.

RP-HPLC chromatograms of phenolic compounds in *J. rigida* needles were analyzed. Based on comparison of retention times with those of the standards, peaks 1, 2, 4, 5, 6, 7, 9, 10, 11, and 13 were identified as chlorogenic acid, catechin, cumaric acid, rutin, hyperoside, ellagic acid, podophyllotoxin, amentoflavone, formononetin, and galangal, respectively (Figure 2, G0). Total contents of the identified compounds varied significantly among the regions (*p* < 0.05, Table 3), the highest recorded in S2 (746.65 mg/g) followed by S6 (694.02 mg/g) and S3 (666.40 mg/g), and the lowest in S11 (415.27 mg/g) and S8 (411.74 mg/g). In all 11 *J. rigida* samples, chlorogenic acid, catechin, podophyllotoxin, and amentoflavone were the most abundant phenolic compounds. The contents of these four compounds varied from 53.32 ± 0.17 to 163.38 ± 0.17 mg/g DW, 112.52 ± 0.08 to 288.88 ± 0.38 mg/g DW, 71.11 ± 0.06 to 189.18 ± 0.04 mg/g DW and 57.68 ± 0.01 to 146.16 ± 0.05 mg/g DW, respectively. Ellagic acid and formononetin were the least abundant phenolic compounds, with contents ranging from 2.82 ± 0.02 to 3.01 ± 0.06 mg/g DW and 2.22 ± 0.01 to 5.68 ± 0.02 mg/g DW, respectively.

**Table 3:** **Contents of 10 phenolic compounds in phenolic extracts of ***J. rigida*** needles from different origins**.

**Compounds**		**Chlorogenic acid**	**Catechin**	**Cumaric acid**	**Rutin**	**Hyperoside**	**Ellagic acid**	**Podophyllotoxin**	**Amentoflavone**	**Formononetin**	**Galangal**	**Total identified**
Content (mg/g)	S1	148.00 ± 0.04^b^	132.05 ± 0.19^j^	4.43 ± 0.02^ab^	7.82 ± 0.04^d^	9.89 ± 0.08^e^	2.92 ± 0.02^a^	142.22 ± 0.03^g^	84.45 ± 0.10^i^	2.91 ± 0.05^i^	22.07 ± 0.06^i^	556.77
	S2	163.38 ± 0.17^a^	288.88 ± 0.38^a^	4.57 ± 0.03^a^	11.51 ± 0.08^a^	11.7 ± 0.03^c^	2.94 ± 0.04^a^	147.71 ± 0.17^d^	88.02 ± 0.16^h^	3.91 ± 0.05^h^	23.98 ± 0.07^h^	746.65
	S3	110.65 ± 0.22^f^	216.59 ± 0.11^c^	4.37 ± 0.03^b^	10.67 ± 0.04^b^	11.93 ± 0.03^b^	2.89 ± 0.01^a^	189.18 ± 0.04^a^	90.55 ± 0.04^g^	4.29 ± 0.06^e^	25.30 ± 0.04^g^	666.40
	S4	64.76 ± 0.14^j^	142.87 ± 0.28^i^	4.41 ± 0.05^ab^	7.12 ± 0.04^e^	11.63 ± 0.06^c^	2.89 ± 0.06^a^	155.92 ± 0.13^c^	134.33 ± 0.15^c^	4.91 ± 0.02^d^	32.96 ± 0.02^b^	561.79
	S5	83.67 ± 0.09^i^	173.99 ± 0.11^f^	4.39 ± 0.04^ab^	6.84 ± 0.03^f^	12.30 ± 0.02^a^	2.88 ± 0.05^a^	147.42 ± 0.19^e^	128.26 ± 0.05^d^	4.10 ± 0.09^f^	27.44 ± 0.06^d^	591.28
	S6	134.19 ± 0.08^d^	158.68 ± 0.17^g^	4.36 ± 0.07^b^	5.22 ± 0.05^g^	10.83 ± 0.05^d^	2.91 ± 0.09^a^	184.81 ± 0.14^b^	152.34 ± 0.16^a^	5.68 ± 0.02^a^	35.00 ± 0.09^a^	694.02
	S7	86.22 ± 0.09^h^	178.38 ± 0.04^e^	4.57 ± 0.04^a^	9.29 ± 0.10^c^	9.49 ± 0.08^g^	2.89 ± 0.04^a^	143.85 ± 0.03^f^	121.87 ± 0.16^e^	5.11 ± 0.02^c^	27.43 ± 0.02^e^	589.11
	S8	53.32 ± 0.17^k^	112.52 ± 0.08^k^	4.37 ± 0.06^b^	4.55 ± 0.04^i^	9.61 ± 0.08*f*^g^	2.82 ± 0.02^a^	77.81 ± 0.05^i^	116.11 ± 0.08^f^	4.06 ± 0.05^g^	26.58 ± 0.07^f^	411.74
	S9	147.74 ± 0.28^c^	279.59 ± 0.18^b^	4.50 ± 0.02^ab^	4.97 ± 0.04^h^	12.14 ± 0.03^a^	3.00 ± 0.07^a^	71.90 ± 0.08^j^	63.26 ± 0.05^j^	2.35 ± 0.04^j^	17.52 ± 0.01^j^	606.97
	S10	116.93 ± 0.29^e^	186.39 ± 0.16^d^	4.43 ± 0.02^ab^	0.53 ± 0.01^k^	9.72 ± 0.04*e*^f^	2.89 ± 0.08^a^	94.92 ± 0.03^h^	146.16 ± 0.05^b^	5.50 ± 0.03^b^	32.63 ± 0.03^c^	600.10
	S11	98.06 ± 0.03^g^	148.25 ± 0.01^h^	4.39 ± 0.04^ab^	2.96 ± 0.05^j^	10.87 ± 0.06^d^	3.01 ± 0.06^a^	71.11 ± 0.06^k^	57.68 ± 0.01^k^	2.22 ± 0.01^j^	16.72 ± 0.03^k^	415.27

*Data (means ± SD, n = 3) within a row with different superscripts are significantly different (p < 0.05)*.

Chlorogenic acid, catechin, podophyllotoxin, and amentoflavone are well-known human health antioxidants (Harwood et al., 2007; Wang et al., 2013). Previous studies have shown that these active components are abundant in *J. rigida* and have significant potential for application as natural antioxidants in the food and drug industries. Since the contents of phenolic compounds in *J. rigida* needles from different geographical sources vary considerably, a standard protocol for effective quality evaluation of *J. rigida* appears essential.

### Antioxidant activity of *J. rigida* needles from different regions

The antioxidant activities of *J. rigida* needles are presented in Table 4. *J. rigida* derived from Yijun (S3) possessed the highest DPPH radical scavenging activity with the lowest mean IC_50_ value (17.99 ± 0.23 μg/mL) while that from Chongli (S5) had the highest mean IC_50_ value (55.17 ± 0.97 μg/mL), indicative of lowest DPPH radical scavenging activity. ABTS activity data were consistent with those of FRAP analysis. ABTS radical scavenging capacity and ferric reducing power of *J. rigida* derived from Yijun (S3, 4862.29 ± 326.73 μmol Trolox/g and 824.28 ± 45.49 μmol Trolox/g, respectively) were also the highest. *J. rigida* derived from Wulahada (S8) displayed the lowest antioxidant activity in both ABTS and FRAP assays with values of 3463.72 ± 102.01 μmol Trolox/g and 467.75 ± 3.61 μmol Trolox/g, respectively. These profiles clearly suggest that the antioxidant ability of *J. rigida* varies between locations, with samples from Fugu (S2) and Yijun (S3) exhibiting the strongest activities.

**Table 4:** **Antioxidant activities of ***J. rigida*** needles from different regions**.

**Origin**	**DPPH IC_50_ (μg/ml)**	**ABTS (μmol Trolox/g)**	**FRAP (μmol Trolox/g)**
S1	26.36 ± 0.80^g^	3768.60 ± 151.91^bc^	561.54 ± 6.04^b^
S2	24.30 ± 0.65^h^	4465.46 ± 210.79^a^	712.19 ± 16.99^a^
S3	17.99 ± 0.23^h^	4862.29 ± 326.73^ab^	824.28 ± 45.49^b^
S4	26.75 ± 0.46^d^	4300.92 ± 171.16^f^	607.94 ± 6.81^ef^
S5	55.17 ± 0.97^f^	4146.06 ± 254.25^de^	623.30 ± 3.49^de^
S6	28.91 ± 0.01^e^	3812.15 ± 65.70^f^	569.38 ± 43.69^ef^
S7	24.01 ± 0.22^b^	4102.51 ± 52.20^ef^	585.07 ± 13.89^f^
S8	30.63 ± 0.35^a^	3463.72 ± 102.01^cd^	467.75 ± 3.61^c^
S9	20.76 ± 0.62^f^	4567.09 ± 26.65^bc^	705.65 ± 13.68^b^
S10	35.65 ± 0.13^e^	3850.87 ± 167.43^cd^	540.95 ± 4.00^cd^
S11	18.69 ± 0.79^c^	4775.18 ± 56.09^g^	727.22 ± 7.64^g^

*Data (means ± SD, n = 3) within a row with different superscripts are significantly different (p < 0.05)*.

These results were consistent with the total contents of 10 phenolic compounds, supporting the theory that active ingredients, such as chlorogenic acid, catechin, podophyllotoxin, and amentoflavone, play important roles in the antioxidant activity of *J. rigida*. The strong antioxidant activities observed for samples from Fugu (S2) and Yijun (S3) may thus be attributed to the high abundance of chlorogenic acid, catechin and podophyllotoxin. In conclusion, variations in the antioxidant activities of plants from different localities are possibly attributable to the contents, properties, and proportions of their phenolic constituents. Furthermore, geographical factors, such as elevation, latitude, longitude, temperature, light, rainfall, and locations of high altitude and cold climatic conditions may be underlying causes of these variations.

### Antibacterial activity of *J. rigida* needles from different regions

The antibacterial activities of phenolic extracts of *J. rigida* were tested against nine strains of bacteria. Two narrow-spectrum antibiotics (penicillin, tetracycline) and one broad-spectrum antibiotic (chloramphenicol) were used as positive controls. DIZ values of phenolic extracts of *J. rigida* against all tested bacteria strains varied from 6.65 to 14.83 mm (Table 5). The largest DIZ was obtained for *K. pneumonia*, followed by *L. monocytogenes, S. paratyphi, S. enteritidis, P. aeruginosa*, and *S. typhimurium*, and the lowest for *S. aurous*. *J. rigida* phenolic extracts exerted antibacterial activity against both gram-positive and -negative bacteria. Among *J. rigida* from the 11 regions, those derived from Fengcheng (S11) and Changbai Mountain (S10) displayed the highest sensitivity against bacteria, with the greatest inhibition zone diameters for *K. pneumonia* (13.03 ± 0.12 mm, 12.17 ± 0.62 mm), followed by *L. monocytogenes* (12.23 ± 0.10 mm, 12.13 ± 0.35 mm), *S. paratyphi* (11.25 ± 0.26 mm, 10.56 ± 0.30 mm), *P. aeruginosa* (12.25 ± 1.35 mm, 10.25 ± 0.35 mm) and *S. typhimurium* (10.87 ± 0.17 mm, 9.00 ± 0.50 mm), clearly indicating strongest antibacterial activity of plants sourced from S11 and S10.

**Table 5:** **Inhibition zone diameter of phenolic extracts of ***J. rigida*** needles from different regions against bacteria**.

**Origin**	**Gram negative**						**Gram positive**		
	***S. typhimurium***	***S. enteritidis***	***S. paratyphi***	***K. pneumoniae***	***P. aeruginosa***	***E. coli***	***B. subtilis***	***L. monocytogenes***	***S. aurous***
S1	9.15 ± 1.63^ef^	10.75 ± 0.35^e^	9.58 ± 0.71^h^	12.43 ± 0.51^d^	8.95 ± 0.13^e^	7.63 ± 0.76^g^	6.67 ± 0.29^i^	12.90 ± 0.14^d^	6.90 ± 0.14^ef^
S2	7.46 ± 0.71^h^	11.53 ± 0.46^d^	6.65 ± 0.52^j^	6.95 ± 0.50^k^	8.87 ± 1.25^e^	13.16 ± 0.28^b^	6.70 ± 0.42^i^	11.23 ± 0.40^h^	6.73 ± 0.29^fg^
S3	9.25 ± 1.56^de^	12.17 ± 0.21^c^	10.87 ± 0.56^f^	12.63 ± 0.95^c^	7.35 ± 0.35^h^	7.72 ± 0.38^fg^	11.35 ± 0.78^c^	13.87 ± 0.21^b^	6.75 ± 0.07^fg^
S4	10.03 ± 0.12^c^	7.66 ± 0.57^f^	9.15 ± 0.14^i^	12.25 ± 0.91^e^	8.13 ± 0.28^g^	10.15 ± 0.21^c^	11.13 ± 0.85^d^	11.90 ± 0.14^f^	7.68 ± 0.20^de^
S5	7.67 ± 0.74^i^	12.65 ± 0.32^b^	14.83 ± 0.25^a^	9.47 ± 0.39^j^	7.05 ± 0.21^i^	14.08 ± 2.67^a^	11.90 ± 1.27^b^	13.18 ± 0.36^c^	6.60 ± 0.24^gh^
S6	9.33 ± 0.29^d^	6.87 ± 0.12^g^	10.63 ± 0.53^g^	10.13 ± 0.36^i^	11.33 ± 0.76^b^	9.60 ± 0.87^d^	8.38 ± 0.48^h^	11.93 ± 0.12^f^	9.93 ± 0.81^a^
S7	12.91 ± 0.17^a^	12.83 ± 0.87^a^	12.41 ± 0.57^b^	13.73 ± 1.25^a^	10.08 ± 0.63^d^	8.53 ± 0.45^e^	9.26 ± 1.26^g^	14.25 ± 1.06^a^	7.23 ± 0.25^c^
S8	10.07 ± 0.12^c^	7.65 ± 0.21^f^	12.23 ± 0.14^c^	10.43 ± 0.12^h^	6.90 ± 0.12^i^	7.67 ± 0.59^fg^	10.50 ± 0.71^f^	11.60 ± 0.36^g^	7.10 ± 0.17^cd^
S9	8.45 ± 0.49^g^	7.65 ± 0.28^f^	11.47 ± 0.35^d^	11.65 ± 0.92^g^	8.45 ± 0.49^f^	6.90 ± 0.14^h^	10.53 ± 1.12^f^	13.85 ± 0.21^b^	7.56 ± 0.52^b^
S10	9.00 ± 0.50^f^	7.66 ± 0.30^f^	10.56 ± 0.30^g^	12.17 ± 0.62^f^	10.25 ± 0.35^c^	7.83 ± 0.25^f^	10.83 ± 0.54^e^	12.13 ± 0.35^e^	6.87 ± 0.12^ef^
S11	10.87 ± 0.17^b^	6.53 ± 0.23^h^	11.25 ± 0.26^e^	13.03 ± 0.12^b^	12.25 ± 1.35^a^	7.55 ± 0.35^g^	13.98 ± 1.27^a^	12.23 ± 0.10^ef^	6.55 ± 0.13^h^
Penicillin	13.08 ± 0.66	7.75 ± 0.96	13.27 ± 0.36	12.37 ± 1.41	22.5 ± 1.87	9.73 ± 0.54	31.85 ± 2.06	10.53 ± 0.50	25.67 ± 0.36
Tetracycline	10.32 ± 1.85	13.55 ± 0.99	14.00 ± 0.41	13.30 ± 0.36	12.45 ± 0.16	12.73 ± 0.59	22.17 ± 1.47	12.02 ± 0.68	16.20 ± 0.25
Chloramphenicol	8.38 ± 1.56	13.00 ± 0.63	12.27 ± 0.64	14.70 ± 0.33	12.30 ± 0.83	8.75 ± 0.29	6.65 ± 0.49	13.5 ± 0.87	7.17 ± 0.29

*Data (means ± SD, n = 3) within a row with different superscripts are significantly different (p < 0.05). Inhibition zone diameter (mm), including sterile disk diameter (6 mm). Penicillin, Tetracycline, and Chloramphenicol were used as the positive controls and DMSO as the negative control*.

MIC and MBC values of the phenolic extracts were determined for further evaluation of *J. rigida* quality (Table 6). The MIC and MBC values for all tested bacterial strains ranged from 0.3125 to 10.00 mg/mL. Consistent with DIZ results, *J. rigida* derived from Fengcheng (S11) and Changbai Mountain (S10) exerted the strongest antibacterial effects with the lowest MIC and MBC values among the 11 *J. rigida* samples examined.

**Table 6:** **MIC and MBC values of phenolic extracts of ***J. rigida*** needles from different regions against bacteria**.

**Bacteria**		**Gram negative**						**Gram positive**
		***S. typhimurium***		***S. enteritidis***	***S. paratyphi***	***K. pneumoniae***	***P. aeruginosa***	***L. monocytogenes***
Origin (mg/ml)	S1	MIC	5	10	0.3125	10	0.3125	0.3125
		MBC	5	10	0.3125	10	0.3125	0.625
	S2	MIC	5	5	0.3125	10	0.3125	0.15
		MBC	5	5	0.3125	10	0.625	0.15
	S3	MIC	1.25	5	0.3125	5	0.3125	0.3125
		MBC	2.5	5	5	5	0.625	0.625
	S4	MIC	2.5	10	0.625	10	0.625	0.625
		MBC	2.5	10	0.625	10	1.25	1.25
	S5	MIC	1.25	1.25	0.3125	10	0.3125	0.3125
		MBC	1.25	1.25	0.3125	10	0.625	0.3125
	S6	MIC	10	10	0.3125	10	0.3125	0.3125
		MBC	10	10	1.25	10	0.3125	0.3125
	S7	MIC	2.5	10	0.625	10	0.625	0.625
		MBC	2.5	10	1.25	10	0.625	0.625
	S8	MIC	2.5	2.5	0.3125	2.5	0.3125	0.3125
		MBC	2.5	2.5	0.625	2.5	0.3125	0.3125
	S9	MIC	10	5	1.25	5	0.3125	0.3125
		MBC	10	5	1.25	5	0.3125	0.625
	S10	MIC	5	5	0.3125	2.5	0.3125	0.3125
		MBC	5	5	0.3125	2.5	0.3125	0.3125
	S11	MIC	5	1.25	0.625	5	0.3125	0.3125
		MBC	5	1.25	0.625	5	0.625	0.625
Positive control (μg/ml)	Penicillin	MIC	2.5	10	10	10	1.25	1.25
		MBC	2.5	10	10	10	1.25	1.25
	Tetracycline	MIC	1.25	2.5	2.5	1.25	0.625	1.25
		MBC	2.5	5	5	2.5	1.25	2.5
	Chloramphenicol	MIC	2.5	2.5	1.25	1.25	2.5	2.5
		MBC	5	2.5	2.5	1.25	5	5

*Penicillin, Tetracycline, and Chloramphenicol were used as the positive controls*.

In conclusion, all the phenolic extracts of *J. rigida* showed broad-spectrum antibacterial activity, with samples from Fengcheng (S11) and Changbai Mountain (S10) exerting the strongest effects. These results were in keeping with the total phenolic and flavonoid contents of *J. rigida*, suggesting that antibacterial activity is closely related to the total phenolic constituent levels. Moreover, since *J. rigida* traditionally achieves its therapeutic effects through strong biological activities, including anti-inflammatory, anticancer, and antiviral properties, antibacterial activity may be considered to possess larger proportion in quality evaluation of *J. rigida* (Gordien et al., 2009; Ryu et al., 2010; Jeong et al., 2012). *J. rigida* derived from Fengcheng and Changbai Mountain were therefore of outstanding quality, supporting their utility as effective potential bacterial inhibitors and bactericidal agents.

### Similarity analysis (SA) of HPLC fingerprints

The *J. rigida* samples collected from 11 different regions were analyzed to develop a standard fingerprint under the established HPLC conditions. Fourteen common peaks were selected as characteristic, and peak 7 (retention time of 30.71 min) as the reference standard peak (Figure 2). Species of the same origin derived from different areas were tentatively identified based on the chromatograms, and similarities assessed with CASES software. The correlation coefficients of the samples ranged from 0.85 to 0.98 (Table 7). A high degree of similarity was detected among Chongli (S5), Dabaozhen (S6), Xiaosuangou (S7), and Wulahada (S8) samples from Hebei province, suggesting that *J. rigida* from Hebei are of the same origin with similar chemical constituents. The chromatograms appeared characteristic of the specific phenolic components, supporting disparities among plants derived from different regions.

**Table 7:** **Similarities of chromatograms of ***J. rigida*** samples based on correlation**.

**No**.	**S1**	**S2**	**S3**	**S4**	**S5**	**S6**	**S7**	**S8**	**S9**	**S10**	**S11**
S1	1.00										
S2	0.966	1.00									
S3	0.959	0.991	1.00								
S4	0.956	0.893	0.888	1.00							
S5	0.974	0.931	0.925	0.99	1.00						
S6	0.947	0.883	0.879	0.989	0.983	1.00					
S7	0.979	0.965	0.963	0.967	0.975	0.955	1.00				
S8	0.947	0.889	0.884	0.989	0.985	0.989	0.963	1.00			
S9	0.92	0.961	0.945	0.848	0.902	0.857	0.905	0.855	1.00		
S10	0.889	0.861	0.855	0.912	0.931	0.946	0.896	0.935	0.909	1.00	
S11	0.951	0.951	0.937	0.917	0.953	0.927	0.935	0.922	0.979	0.95	1.00

### Hierarchical clustering analysis (HCA)

**Figure 3:**
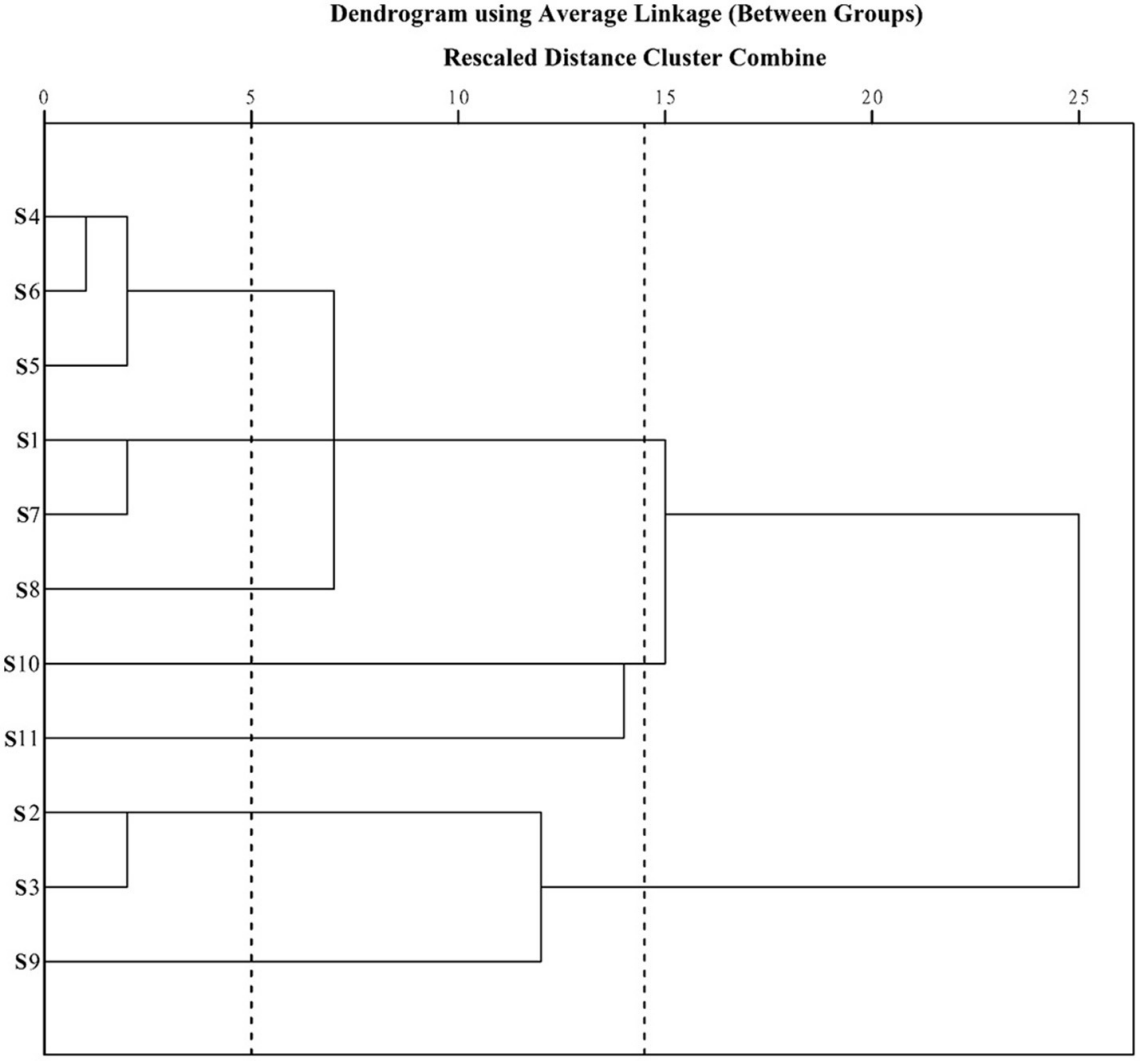
**Dendrograms of hierarchical cluster analysis (HCA) for samples of ***J. rigida*****.

Prior to HCA, data were visually analyzed via overlapping tests using ChemStation software (Agilent, USA), and divided into three distinct patterns (G1, G2, and G3; Figure 3). Due to the subjective and non-quantitative nature of visual comparisons, the HCA assay was selected for quantitative assessment of *J. rigida* and relative peak areas (RPA) of the 14 common chromatographic peaks determined. A 14 × 11 matrix was formed for HCA using SPSS software, and a dendrogram acquired using average linkage between groups and the cosine method (Figure 3). The 11 samples were assorted into three quality clusters assuming a proper level of distance (14.5), consistent with the subjective results of visual classification. Group 1 included *J. rigida* derived from Fugu (S2), Yijun (S3), and Jinhua (S9) while Group 2 contained *J. rigida* from Fengcheng (S11) and Changbai Mountain (S10). Samples from the other six regions, including Helan Mountain (S1), Datong (S4), Chongli (S5), Dabaozhen (S6), Xiaosuangou (S7), and Wulahada (S8), were clustered in Group 3 owing to similarities in their chemical constituents. Group 2 possessed the highest contents of phenolic compounds while Group 1 exhibited the highest antioxidant activity. The quality of *J. rigida* derived from distinct regions differed due to a number of factors, such as altitude, latitude, longitude, and soil nutrients. Correlation coefficients of chromatograms within Groups 1, 2, and 3 corresponding to the simulated mean chromatograms were generated from the software.

The correlation coefficients of each chromatogram for G1, G2, and G3 are presented in Table 7. Chromatograms were similar within a particular group while significantly distinct between groups. The differences in similarity values for the three groups were consistent with data generated from HCA (Table 8). These results illustrate that HCA can effectively distinguish between *J. rigida* samples of the same origin from different districts and confirm classification based on morphological characteristics (Table 1).

**Table 8:** **Correlation coefficients between individual chromatograms within a group and group simulative mean chromatogram and between group simulative mean chromatograms**.

**Group**	**G1**	**G2**	**G3**
G1	0.942 ± 0.032^a^ (*n* = 3)	0.858^b^	0.846^b^
G2		0.905 ± 0.005^a^ (*n* = 2)	0.893^b^
G3			0.946 ± 0.034^a^ (*n* = 6)

a *Correlation coefficient of individual chromatograms to the simulative mean chromatogram of the corresponding group. Values are presented as means ± SD*.

b *Correlation coefficient between simulative mean chromatograms*.

### Principal component analysis (PCA)

**Figure 4:**
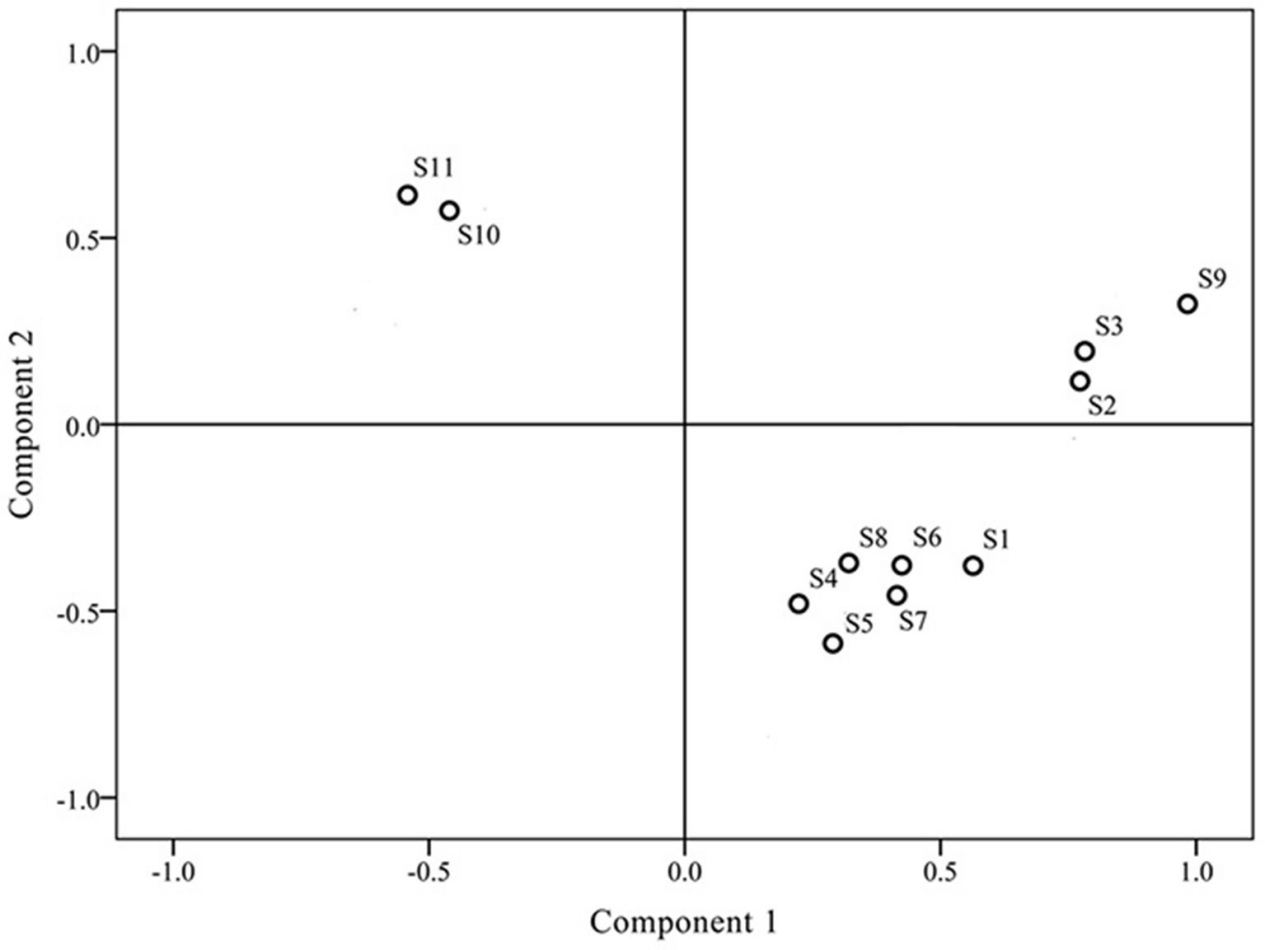
**Scores plot generated from principal component analysis (PCA) of all ***J. rigida*** samples**.

**Figure 5:**
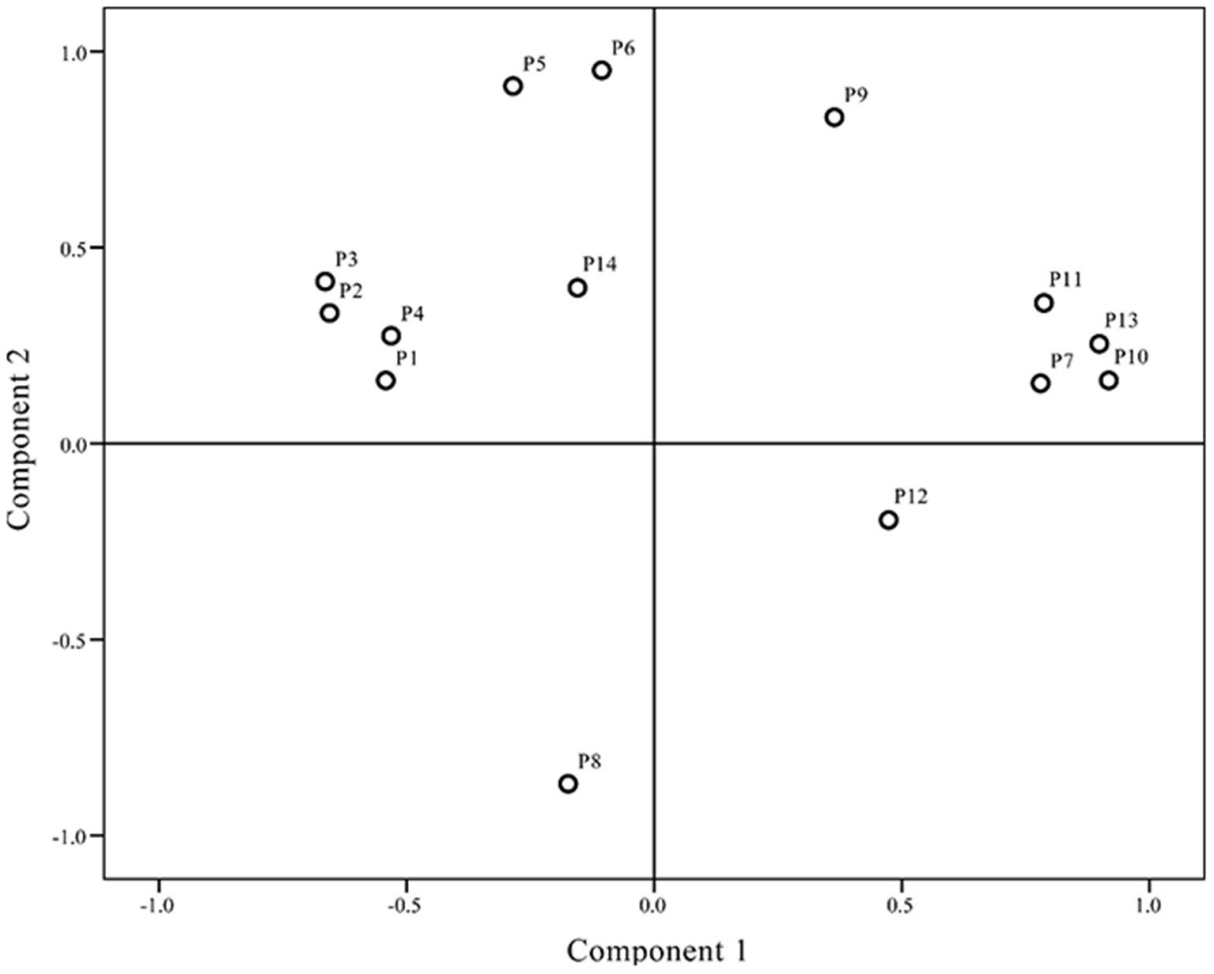
**Scores plot generated from principal component analysis (PCA) of variables (peaks 1–14)**.

Differences in chromatograms of samples mainly existed due to content variations in the common components. To evaluate the discrimination capacity of the common constituents, PCA was conducted using the RPAs of common peaks using HCA input data. The first two principal components contained the most information of all variables, accounting for 87.51% of total variability. The score plot of the first three principal components, PC1 and PC2, visually revealed a positive influence on quality evaluation of *J. rigida* from different regions (Figure 4). PCA results were consistent with HCA findings, indicative of different chemical profiles from distinct locations. Analysis of the loading plot of PC1 against PC2 (Figure 5) revealed that peaks contributing to PC1 (7, 9, 10, 11, and 13) influenced the cluster in a top-down order. These were the compounds that distinguished S2, S3 and S9 as Group 1. Peaks 1, 2, 3, 4, 5, 6, and 14 that mainly contributed to PC2 led to the classification of S10 and S11 as Group 3. The remaining samples comprised Group 2. In summary, since PC1 contributing more peaks than PC2, samples on the right side of Figure 5 were presumed to be of higher quality. Accordingly, we concluded that *J. rigida* collected from Shaanxi province (S2, S3) and Jinhua (S9) were of the highest quality. Data from HCA and PCA supported each other and validated the quality evaluation of *J. rigida* samples.

### Discriminant analysis (DA)

Discriminant analysis was employed to establish a predictive model to facilitate clustering and distinguishing of group members based on observed characteristics. The discriminant function generated on the basis of linear combinations of predictor variables was used to further discriminate and classify the unknown members. These functions were generated from samples with known membership and could be applied to new cases with known values for predictor variables but unknown group membership variables of unknown group membership.

Although 14 peaks were selected as variables from the patterns, not all the variables generated contributed to development of the discriminant function. Only valuable predictor variables may be selected to generate discriminant functions using this procedure. Two types of discriminant functions were acquired using SPSS software.

Canonical discriminant function:

$$\begin{array}{l} Y_{1}=0.007{\text{X}}_{1}-0.026{\text{X}}_{2}+0.005{\text{X}}_{3}+0.026{\text{X}}_{4}-0.0.002{\text{X}}_{5}\\ +\;0.003{\text{X}}_{7}+7.511\\ Y_{2}=-0.005{\text{X}}_{1}+0.012{\text{X}}_{2}-0.004{\text{X}}_{3}+0.007{\text{X}}_{4}+0.001{\text{X}}_{5}\\ +\;0.002{\text{X}}_{6}+0.001{\text{X}}_{7}-2.406 \end{array}$$

Discrimination standard:

$$\begin{array}{l} {\text{Y}}_{1}>\;0\text{ and }{\text{Y}}_{1}\;>{\text{Y}}_{2}:\;{\text{G}}_{1}\\ {\text{Y}}_{1}<\;0\text{ and }{\text{Y}}_{1}\;<{\text{Y}}_{2}:\;{\text{G}}_{2}\\ {\text{Y}}_{2}<\;0\text{ and }{\text{Y}}_{1}\;<{\text{Y}}_{2}:\;{\text{G}}_{3} \end{array}$$

Fisher's discrimination function:

$$\begin{array}{l} G_{1}=-1.24{\text{X}}_{1}+0.468{\text{X}}_{2}-0.081{\text{X}}_{3}-0.468{\text{X}}_{4}+0.042{\text{X}}_{5}\\ -\;0.005{\text{X}}_{6}-0.051{\text{X}}_{7}-203.055\\ G_{2}=-0.008{\text{X}}_{1}+0.038{\text{X}}_{2}+0.009{\text{X}}_{3}-0.057{\text{X}}_{4}+0.009{\text{X}}_{5}\\ -\;0.014{\text{X}}_{6}-0.005{\text{X}}_{7}-34.659\\ G_{3}=-0.023{\text{X}}_{1}+0.059{\text{X}}_{2}-0.006{\text{X}}_{3}-0.057{\text{X}}_{4}+0.009{\text{X}}_{5}\\ -\;0.004{\text{X}}_{6}+0.003{\text{X}}_{7}-36.699 \end{array}$$

**Figure 6:**
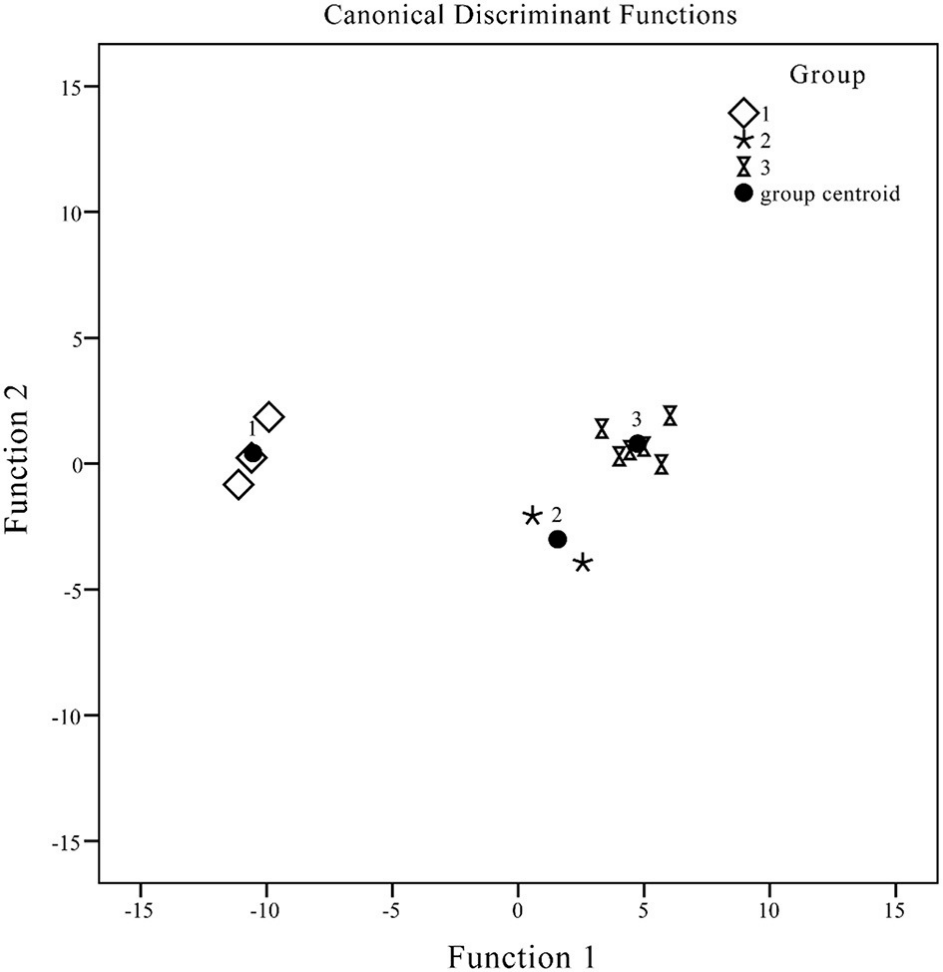
**Canonical discrimination analysis (DA) of HPLC chromatograms for ***J. rigida*** samples**.

Standard for discriminant: each sample was assigned to a group according to the highest of the three functional values. X represents the variable and G1, G2, and G3 denote samples from Groups 1, 2, and 3, respectively. The variables assigned to the areas of peaks 1–7 were adopted to develop the discriminant functions. The inputted values of the three variables were obtained to form discriminant standard values and classify unknown samples. High-resolution DA plots for the three groups are displayed in Figure 6. The seven variables employed were the most discriminating, so that analytes included in Groups 1, 2, and 3 could be divided with 100% accuracy.

The phenolic components differed significantly among different regions of origin of *J. rigida* according to HPLC fingerprint analysis. These results were consistent with SA, HCA, PCA, and DA data, clearly indicating that the techniques were effective for quality assessment of *J. rigida*. The quality of medicinal herbs may contribute to the integrated influence of composite factors, such as environmental or genetic factors, cultivation methods, and collection periods. Therefore, quality evaluation of *J. rigida* via HPLC fingerprint analysis combined with chemometrics methods may be of vital importance to optimize its future use. Further studies should focus on the collection of samples from various regions of China and other countries for analysis via HPLC fingerprint combined with chemometrics as well as examination of environmental and genetic factors.

## Conclusions

HPLC fingerprinting coupled with chemometrics was developed for the quality evaluation of *J. rigida* collected from different regions of China. All the *J. rigida* samples investigated possessed distinct phenolic profiles (based on assessment of total phenolic and total flavonoid contents and 10 phenolic compounds) and bioactivities, leading to two main conclusions. Firstly, *J. rigida* samples derived from Fengcheng (S11) and Changbai Mountain (S10) were of the highest quality in terms of effective bacterial inhibitor and bactericidal activities, attributable to their high total phenolic and total flavonoid contents and antibacterial effects. Secondly, *J. rigida* samples from Jinhua (S9) and Shaanxi (S2, S3) contained the most active ingredients (10 phenolic compounds) and showed the strongest antioxidant activities. These results provide critical information for quality evaluation of *J. rigida* samples from different geographical regions. Integrated information from comparisons and analyses revealed that samples of *J. rigida* collected from Jilin and Liaoning province displayed the highest bioactivity, possibly attributable to the geographical location at high altitudes and cold climatic conditions, followed by samples from Shaanxi province.

Combinations of RP-HPLC fingerprint SA, HCA, PCA, and DA were further adopted to develop an effective method for accurate classification and quantification of *J. rigida*. Fourteen characteristic peaks were identified, and a standard fingerprint validated for screening and quality evaluation of *J. rigida*. Furthermore, sample grouping based on HCA and PCA analyses coincided with their geographical regions of origin. Data from DA analysis further facilitated the development of a simple and reliable method with high precision, stability, and repeatability that could be adopted for accurate classification and quality control of samples of *J. rigida* with unknown membership.

In conclusion, the study have developed a flexible method involving HPLC fingerprint combined with chemometrics to evaluate the quality of traditional Chinese medicine. The accurate classification of *J. rigida* represents an essential first step in creating a validation system for the development of *J. rigida*-based health foods and supplements, which should increase consumer confidence in the efficacy and safety of the products and help manufacturers meet current and future quality criteria set by the regulatory authorities. Further research is warranted to clarify the relationship between environmental factors and phytochemical variations of *J. rigida* from different regions.

## Author contributions

DW and DL conceived and designed the experiments. ZL and SZ performed the experiments and analyzed the data. ZL, DW, and DL wrote the paper. DW critically revised the paper.

### Conflict of interest statement

The authors declare that the research was conducted in the absence of any commercial or financial relationships that could be construed as a potential conflict of interest.
